# Kunjin Virus, Zika Virus, and Yellow Fever Virus Infections Have Distinct Effects on the Coding Transcriptome and Proteome of Brain-Derived U87 Cells

**DOI:** 10.3390/v15071419

**Published:** 2023-06-23

**Authors:** Carolin Brand, Gabrielle Deschamps-Francoeur, Kristen M. Bullard-Feibelman, Michelle S. Scott, Brian J. Geiss, Martin Bisaillon

**Affiliations:** 1 Département de Biochimie et de Génomique Fonctionnelle, Faculté de Médecine et des Sciences de la Santé, Université de Sherbrooke, 3201 rue Jean-Mignault, Sherbrooke, QC J1E 4K8, Canada; carolin.brand@usherbrooke.ca (C.B.); gabrielle.deschamps-francoeur@usherbrooke.ca (G.D.-F.); michelle.scott@usherbrooke.ca (M.S.S.); 2 Department of Microbiology, Immunology, and Pathology, School of Biomedical Engineering, Colorado State University, 1682 Campus Delivery, Fort Collins, CO 80523, USA; feibelman.k@gmail.com (K.M.B.-F.); brian.geiss@colostate.edu (B.J.G.)

**Keywords:** flavivirus, Kunjin virus, Zika virus, yellow fever virus, virus–host interaction, transcriptomics, alternative splicing, proteomics, RNA-Seq, SILAC

## Abstract

As obligate intracellular parasites, viruses rely heavily on host cells for replication, and therefore dysregulate several cellular processes for their benefit. In return, host cells activate multiple signaling pathways to limit viral replication and eradicate viruses. The present study explores the complex interplay between viruses and host cells through next generation RNA sequencing as well as mass spectrometry (SILAC). Both the coding transcriptome and the proteome of human brain-derived U87 cells infected with Kunjin virus, Zika virus, or Yellow Fever virus were compared to the transcriptome and the proteome of mock-infected cells. Changes in the abundance of several hundred mRNAs and proteins were found in each infection. Moreover, the alternative splicing of hundreds of mRNAs was found to be modulated upon viral infection. Interestingly, a significant disconnect between the changes in the transcriptome and those in the proteome of infected cells was observed. These findings provide a global view of the coding transcriptome and the proteome of Flavivirus-infected cells, leading to a better comprehension of Flavivirus–host interactions.

## 1. Introduction

Viruses are obligate intracellular parasites that are dependent on host cells for replication. During infection, viruses modulate several cellular processes for their benefit. In response, host cells activate multiple signaling pathways to restrict viral replication and eliminate the pathogen. Innate immune sensors, also called pattern recognition receptors (PRRs), recognize pathogen-associated molecular patterns (PAMPs), such as viral nucleic acids and proteins, and activate signaling cascades to induce antiviral and proinflammatory genes, including interferons. Interferons then promote the expression of interferon-stimulated genes (ISGs), the protein products of which have antiviral effector functions [1]. One example of an interferon-induced gene is protein kinase R (PKR). PKR is activated upon interaction with viral double-stranded RNA, phosphorylates eIF2α, and thus blocks the translation of both viral and host mRNAs [2] by sequestering stalled translation preinitiation complexes in stress granules.

Flaviviruses are mosquito-borne viruses with an 11 kb, positive sense, single-stranded RNA genome [3]. They are globally distributed, putting more than half of the world’s population at risk for infection. Although most Flavivirus infections are asymptomatic and the most common clinical manifestation is a flu-like illness, Flaviviruses can cause a wide variety of severe diseases, such as encephalitis, hemorrhagic fever, or jaundice in a small percentage of cases. Members of the Flavivirus genus include the important human pathogens Yellow Fever virus (YFV), Zika virus (ZIKV), West Nile virus (WNV), Dengue virus (DENV), and Japanese Encephalitis virus (JEV) [4,5]. When transmitted by a mosquito vector, Flaviviruses infect human skin cells, such as skin dendritic cells. These cells then migrate to lymphoid organs, from where the virus can be disseminated throughout the body including the central nervous system where they can cause life-threatening encephalitis [6].

Upon infection, Flaviviruses dysregulate a multitude of cellular processes for their benefit. For example, DENV has been shown to increase fatty acid biosynthesis by recruiting fatty acid synthase (FASN) to the sites of viral replication and stimulating FASN activity [7]. This contributes to the alteration of the lipid profile of DENV-infected cells, affecting membrane properties and promoting the formation of viral replication compartments [8]. Flaviviruses also counteract measures taken by the host cell to limit viral replication. For instance, they have been shown to inhibit interferon signaling [1] as well as the formation of stress granules [9,10] to evade the antiviral response and overcome the translational block of viral RNA, respectively. Moreover, recent studies have shown that Flaviviruses alter the host cell’s transcriptome by affecting gene expression levels and pre-mRNA splicing [11,12,13,14,15]. For example, DENV has been shown to affect SAT1 splicing patterns. More precisely, an increase in exon 4 inclusion in SAT1 mRNA has been observed in DENV-infected cells, leading to the degradation of SAT1 mRNA, and therefore to a reduction in the amount of the antiviral protein SAT1 [16].

Most of the transcriptomic profiling studies following infection with Flaviviruses have been performed with a single viral species, and experimental conditions between different studies vary greatly, preventing the comparison of changes induced in the host cell transcriptome by these closely related viruses. Here, to gain insight into the range of molecular effects Flavivirus infections bring about in host cells, we investigate the changes in the coding transcriptome and the proteome of host cells upon infection with three different Flaviviruses (Kunjin, Zika, and Yellow Fever) using next-generation RNA sequencing and Stable Isotope Labeling by Amino acids in Cell culture (SILAC), respectively.

## 2. Materials and Methods

### 2.1. Viruses

Kunjin virus (strain FLSDX, GenBank AY274504.1), Zika virus (strain PRVABC59, GenBank, KU501215.1), Yellow Fever virus (strain 17D, GenBank X03700.1), and Sindbis virus (strain TE3′2J, NCBI reference sequence NC_001547.1) were used for viral infections. To generate viral stocks, VeroE6 cells were grown in DMEM (Wisent Bioproducts, St-Jean-Baptiste, QC, Canada, 319-015-CL) supplemented with 10% FBS (Wisent 081-110) and 50 mM HEPES (Wisent 330-050-EL) to 50% confluency. Viruses were added to the cells for 72 h, infected media were collected, clarified by centrifugation at 15,000 rpm, and frozen at −80 °C. Viral titers were determined using focus-forming assays (KUNV, YFV, ZIKV) or plaque assay titrations (SINV) on VeroE6 cells. VeroE6 cells seeded on 12-well tissue culture plates (250,000 cells per well) were infected with serial dilutions of virus samples for 1 h at 37 °C, and then an agarose nutrient overlay was added (1.5 mL per well; 50% *v*/*v* mix of 2X DMEM (Wisent 319-205-CL) supplemented with 5% FBS and 0.6% agarose). Cells were maintained at 37 °C for 3 days, and on day 3, cells were fixed by adding 1 mL of 10% formaldehyde (Fisher Scientific, Waltham, MA, USA, BP531-500) for 30 min. For the plaque assay (SINV), fixed cells were treated with 1% crystal violet in 20% ethanol for 15 min. The stain was washed off with water, viral plaques were counted, and viral titers were determined as plaque forming units (PFU)/mL. For the focus-forming assay (Flaviviruses), fixed cells were washed with PBS prior to incubation with monoclonal anti-flavivirus group antigen (clone D1-4G2-4-15) antibody at 500 ng/mL in PBS/0.3% Tween 20/1 mg/mL BSA for 16 h at 4 °C. Cells were washed with PBS/0.5% Tween 20 (PBS-T), incubated with anti-mouse HRP antibody (NEB, Ipswitch, MA, USA, 7076), diluted 1:5000 in PBS/0.3% Tween 20/1 mg/mL BSA for 2 h at room temperature, washed with PBS-T, and treated with 3,3′,5,5′-tetramethylbenzidine (TMB, Sigma-Aldrich, St. Louis, MO, USA, 613548) for 30 min. Spots were counted and viral titers determined as focus-forming units (FFU)/mL.

### 2.2. Cell Culture and Infections for RNA Sequencing

U87 cells were grown in complete Dulbecco’s modified Eagle’s Medium (cDMEM) containing 10% fetal bovine serum (Atlas Biologicals, Fort Collins, CO, USA), and L-glutamine, and cells were incubated at 37 °C in humidified incubators supplemented with 5% CO_2_. To perform infections, 50% confluent U87 cells in T150 flasks were either infected at a multiplicity of infection (MOI) of 5 or treated with cell-conditioned media (*n* = 3 for each virus and control). Then, 24 h post-infection, cells were harvested and homogenized using QIAshredder (Qiagen, Hilden, Germany).

### 2.3. Next Generation RNA Sequencing

RNA was isolated using the RNeasy kit (Qiagen) following the manufacturer’s recommended protocols. RNA was stabilized for storage using an RNA transport kit (OMEGA bio-tek, Norcross, GA, USA, R0527). PolyA-RNA was purified from 5 μg total RNA using NEB magnetic mRNA Isolation Kit (NEB, Ipswitch, MA, USA, #S1550S) and eluted into a final volume of 25 μL. Sequencing libraries were prepared using 9 μL of isolated mRNA and ScriptSeq RNA-Seq Library Preparation Kit (Epicentre Biotechnologies, Madison, WI, USA, SSV21124). Paired-end 100 bp sequencing was performed on a HiSeq 4000 system (Illumina, San Diego, CA, USA) at McGill University and Génome Québec Innovation Centre, obtaining between 34 and 72 million reads per sample.

### 2.4. Building of the Reference Genome for RNA Sequencing Analysis

The human genome as well as an annotation file (GRCh38, release 89) were obtained from Ensembl. The viral genome sequences were obtained from NCBI (AY274504.1 for Kunjin virus, KU501215.1 for Zika virus, X03700.1 for Yellow Fever virus, NC_001547.1 for Sindbis virus). A reference genome containing the human genome as well as the viral genomes was generated with STAR 2.5.1b [17] and the parameter --sjdbOverhang 99.

### 2.5. Differential Gene Expression Analysis

Reads were trimmed using Trimmomatic 0.32 [18] to remove adapter sequences as well as nucleotides with low quality scores (TRAILING: 30). The quality of output reads was verified using FastQC 0.11.5. Reads were aligned to the reference genome (hg38 + viral genomes) using STAR 2.5.1b [17] with default parameters except for --outFilterMismatchNmax 5. The numbers of raw reads and aligned reads are given for each sample in Supplementary Figure S2. Aligned reads were then assigned to genes and quantified using Rsubread.FeatureCounts 1.20.6 with options isGTFAnnotationFile = TRUE, countChimericFragments = FALSE, largestOverlap = TRUE, isPairedEnd = TRUE, useMetaFeatures = TRUE, requireBothEndsMapped = TRUE, strandSpecific = 1, minOverlap = 10, autosort = TRUE. Differential gene expression analysis was performed with DESeq2 [19] using default parameters.

### 2.6. Differential Alternative Splicing Analysis

As required for downstream analysis, all reads were trimmed to the same length using Trimmomatic 0.32 [18] before being aligned to the reference genome using STAR 2.5.1b [17]. Alternative splicing analysis was performed using rMATS 3.2.5 [20] with the options -len 93 -a 3 -t paired -analysis U. ΔPSI (percent spliced in) values for each alternative splicing event were calculated by subtracting the PSI value in infected cells from the PSI value in non-infected cells. Therefore, a positive ΔPSI value indicates an increase in the short form upon infection, whereas a negative ΔPSI value indicates an increase in the long form upon infection.

### 2.7. RT-qPCR

Twenty-one genes for which their expression was significantly altered upon viral infection, as determined by RNA sequencing, were selected for RT-qPCR validation: ATOH8, BMPER, CSF2, CXCL8, DDIT3, DGAT2, ENG, ERN1, HBEGF, HERPUD1, IFIT2, IGFL3, IL6, IL7R, INSIG1, MSC, PLAU, RSAD2, TGOLN2, TNFRSF11B, and VAV2. This selection represents genes associated with a varying range of fold-change values, some of which are positive (overexpressed upon infection), and some of which are negative (repressed upon infection). Some of the selected genes are overexpressed or repressed in multiple infections, whereas others are associated with significant fold-change values in one infection only. Reverse transcription reactions were performed on 2.2 μg of total RNA using Transcriptor reverse transcriptase, random hexamers, dNTPs (Roche Diagnostics, Rotkreuz, Switzerland), and 10U RNaseOUT (Invitrogen, Waltham, MA, USA, #10777019), according to the manufacturer’s protocol in a total volume of 20 μL. All gene-specific primers (IDT) were resuspended individually to 20–100 μM in TE buffer (IDT) and then diluted as forward/reverse primer pairs in nuclease-free water (IDT) to a final concentration of 1 μM. Quantitative PCR was performed with 5 μL iTaq Universal SYBR Green Supermix (Bio-Rad Laboratories, Hercules, CA, USA), 2 μL primer pair solution, and 3 μL (10 ng) cDNA in a total volume of 10 μL on a CFX-96 thermocycler (Bio-Rad). After an initial denaturation at 95 °C for 3 min, 50 three-step cycles (denaturation at 95 °C for 15 s, annealing at 60 °C for 30 s, and elongation at 72 °C for 30 s) were executed. For each primer pair, a negative control reaction without a template was performed. Relative expression levels were calculated using the qBASE framework [21].

### 2.8. ASPCR (Alternative Splicing Analysis by End-Point RT-PCR)

Six alternative splicing events that were significantly altered upon viral infection, as determined by RNA sequencing, were selected for ASPCR validation. Primers were designed to anneal on both sides of the alternative splicing event to be analyzed, thus allowing the amplification of both isoforms in the same reaction. RNA integrity was assessed with an Agilent 2100 Bioanalyzer (Agilent Technologies, Santa Clara, CA, USA). Reverse transcription was performed on 1.1 μg total RNA with Transcriptor reverse transcriptase, random hexamers, dNTPs (Roche Diagnostics, Rotkreuz, Switzerland), and 10 units of RNAseOUT (Invitrogen, #10777019) following the manufacturer’s protocol in a total volume of 10 μL. All forward and reverse primers were individually resuspended to 20–100 μM in Tris-EDTA buffer (IDT) and diluted as a primer pair in RNase DNase-free water (IDT) to a final concentration of 1.2 μM. End-point PCR reactions were carried out on 10 ng cDNA in a 10 μL final volume containing 0.2 mmol/L of each dNTP, 0.6 μmol/L of each primer, and 0.2 units of TransStart FastPfu Fly DNA Polymerase (TransGen Biotech, Beijing, China). An initial incubation of 2 min at 95 °C was followed by 35 cycles of denaturation at 95 °C for 20 s, annealing at 55 °C for 20 s, and elongation at 72 °C for 60 s. The amplification was completed by a 5 min incubation at 72 °C. PCR reactions were carried out on thermocyclers C1000 Touch Thermal cycler (Bio-Rad Laboratories, Hercules, CA, USA), and the amplified products were analyzed by automated chip-based microcapillary electrophoresis on Labchip GX Touch HT instruments (Perkin Elmer, Waltham, MA, USA). Amplicon sizing and relative quantitation was performed by the manufacturer’s software.

### 2.9. Cell Culture and Infections for SILAC

U87 cells were grown in SILAC DMEM Flex Media (Life Technologies, Carlsbad, CA, USA, A2493901) supplemented with Glutamax, 50 mM HEPES (pH 7.5), glucose, 10% dialyzed fetal bovine serum, as well as light/medium/heavy L-arginine (R0/R6/R10) and L-lysine (K0/K4/K8). Cells were incubated at 37 °C in humidified incubators supplemented with 5% CO_2_. Cells were passaged by scraping in a cell dissociation buffer (Thermo Fisher Scientific 13151014) instead of using trypsin to maintain isotope-specific labeling. To perform infections, 50% confluent U87 cells in T150 flasks were either infected at a multiplicity of infection (MOI) of 3 or treated with cell-conditioned media (*n* = 3 for each virus and control). Then, 24 h post-infection, cells were scraped from the flask, pelleted, and resuspended in 1 ml lysis buffer (50 mM Tris-Base pH 7.4, 1 mM EDTA, 150 mM NaCl, 1% NP40, 0.25% sodium deoxycholic acid, 1 tablet of cOmplete Protease Inhibitor Cocktail (Roche 05892791001) per 50 mL).

Viruses used for the SILAC experiment were grown as described above, but in cell culture media containing medium/heavy L-arginine (R6/R10) and L-lysine (K4/K8).

### 2.10. Gel Electrophoresis, in-Gel Digestion, and Extraction of Peptides

The protein concentration of each cell lysate was determined using a BCA assay (Pierce, Waltham, MA, USA, 23223), and equal amounts of light, medium, and heavy lysates were mixed (15 μg of each sample for L:M:H mix, and 25 μg of each sample for L:M mix, final volume of approximately 40 μL for each mix). Samples were reduced by adding 0.5 μL of 1M dithiothreitol (DTT) and 8 μL of 5× Laemmli buffer, followed by an incubation at 95 °C for 2 min. Proteins were alkylated by adding 2.5 μL of 1 M iodoacetamide and incubating at room temperature in the dark for 30 min prior to separation by one-dimensional SDS-PAGE (4–12% Bis-Tris Novex mini-gel, Life Technologies, Carlsbad, CA, USA) and visualization by Coomassie Blue staining (Simply Blue Safe Stain, Life Technologies). Following extensive washes in water, each lane of the gel was cut into five slices, and each slice was cut into small pieces with a clean scalpel. To de-stain the gel bands, they were washed four times for 15 min in a shaker. The first wash was with water (Fluka Analytical, Buchs, Switzerland, 39253), and for the second wash, an equal volume of acetonitrile (CH_3_CN, Fluka Analytical 34967) was added. For the third wash, the supernatant was removed before adding 20 mM ammonium bicarbonate (NH_4_HCO_3_), and for the fourth wash, the supernatant was discarded and a 50% *v*/*v* mix of 20 mM NH_4_HCO_3_ and 100% CH_3_CN was added to the gel bands. To dehydrate the gel band pieces, the supernatant from the last wash was removed and 150 μL acetonitrile was added, followed by incubation at room temperature in a shaker for 5 min twice. Finally, the pieces were dried in a speed vacuum centrifuge at 60 °C for 15 min.

Proteins were digested by adding 75 μL of 12.5 ng/mL trypsin (Promega, Madison, WI, USA, V5280) in 20 mM NH_4_HCO_3_ at 30 °C for 16 h. For the extraction of peptides from the gel band pieces, an equal volume of CH_3_CN was added, followed by a 30 min incubation at 30 °C, and the supernatant was transferred to a LoBind tube (Eppendorf, Hamburg, Germany). To further extract peptides, the gel band pieces were incubated at room temperature on a shaker with 75 μL of 1% formic acid (FA, Fisher Scientific, Waltham, MA, USA, A11750) for 20 min (twice), and with 150 μL CH_3_CN for 10 min (three times). All supernatants were pooled in the same LoBind tube. The solvent was removed by lyophilization in a speed vacuum centrifuge at 60 °C for 5 h, and the tryptic peptides were resuspended in 30 μL of 0.1% trifluoroacetic acid (TFA, Sigma Aldrich, St. Louis, MO, USA, T6508) and cleaned up using a ZipTip (Millipore, Burlington, MA, USA, ZTC18S960). The resin was wetted with 100% acetonitrile and equilibrated with 0.1% TFA before the peptides were aspirated. The peptides were then washed with 0.1% TFA and eluted with 1% FA/50% acetonitrile. The clean peptides were dried in a speed vacuum centrifuge at 60 °C for 75 min before being resuspended in 25 μL of 1% formic acid.

### 2.11. LC-MS/MS

A Dionex Ultimate 3000 nanoHPLC system was used to separate 2 μg of trypsin-digested peptides. The sample was loaded onto a Trap column (Acclaim PepMap 100 C18 column (0.3 mm id × 5 mm, Dionex Corporation, Sunnyvale, CA, USA)) with a constant flow of 4 μL/min. Peptides were eluted with a linear gradient of 5–35% solvent B (90% acetonitrile with 0.1% formic acid) onto an analytical column (PepMap C18 nano column (75 mm × 50 cm, Dionex Corporation) over 4 h with a constant flow of 200 nL/min. An EasySpray source was set to 40 °C and a spray voltage of 2.0 kV was used to connect the nanoHPLC system to an OrbiTrap QExactive mass spectrometer (Thermo Fisher Scientific, Waltham, MA, USA). Full scan MS survey spectra (*m*/*z* 350–1600) were acquired in profile mode at a resolution of 70,000 after the accumulation of 1,000,000 ions. The ten most intense peptide ions from the preview scan were selected for fragmentation by collision induced dissociation (CID) with a normalized energy of 35% and a resolution of 17,500 after the accumulation of 50,000 ions, with a filling time of 250 ms and 60 ms for the full scans and the MS/MS scans, respectively. The screening of the precursor ion charge state was enabled, and all unassigned charge states as well as single, 7, and 8 charged species were rejected. A dynamic exclusion list was allowed for a maximum of 500 entries, with a relative mass window of 10 ppm and a retention time of 60 s. The lock mass option was enabled for survey scanning to improve mass accuracy. Data were acquired using the Xcalibur software version 4.3.73.11 (Thermo Fisher Scientific, Waltham, MA, USA).

### 2.12. Peptide Quantification and Differential Protein Expression Analysis

Peptide identification and quantification were performed using the MaxQuant software package version 1.6.14, with the protein database from Uniprot (*Homo sapiens*, 16 July 2013, 88,354 entries). The evidence.txt output file was used for differential protein expression analysis with the Proteus R package 0.2.15 (default parameters).

### 2.13. Venn Diagrams

Venn diagrams were created using InteractiVenn [22] and/or BioVenn [23]. The statistical significance of the overlaps was calculated using a Fisher’s Exact Test available at https://www.langsrud.com/fisher.htm (accessed on 10 May 2022).

### 2.14. Correlation Coefficients

Nonparametric Spearman correlation matrices were computed using GraphPad Prism with default parameters.

### 2.15. Gene Ontology Enrichment Analysis

Gene ontology (GO) enrichment analysis was performed using the Database for Annotation, Visualization, and Integrated Discovery (DAVID) 6.8 [24]. The background for the enrichment analysis included all genes detected in the experimental condition (i.e., all genes in the DESeq2 or rMATS output files), whereas the gene list included only genes that were significantly different between infected and control cells, regarding gene expression levels or alternative splicing. In the Functional Annotation Tool, GOTERM_BP_DIRECT in the section “Gene Ontology” was chosen. Obtained *p* values were subjected to Bonferroni correction.

### 2.16. Western Blot

Two of the most downregulated proteins in Flavivirus infections, as determined in the SILAC experiment, were selected for validation by Western blot: HMGA1 and MRPS27. In total, 1 μg (HMGA1) and 10 μg (MRPS27) of total protein extract were separated by one-dimensional SDS-PAGE at 180 V for approximately 1 h before being transferred to a PVDF blotting membrane (GE 10600023) at 100 V for 1 h 15 min. After blocking in 2.5% non-fat milk in PBS for 1 h, the membranes were incubated with a 1:3000 dilution of primary antibody (anti-HMGA1: Abcam ab129153; anti-MRPS27: Abcam ab153940) for 6 h at room temperature, washed three times in TBS-T for 5 min, incubated with a 1:6000 dilution of anti-rabbit HRP secondary antibody (Abcam, Cambridge, UK, ab205718), washed twice in TBS-T and once in PBS for 5 min. Finally, ECL substrate (Bio-Rad Laboratories, Hercules, CA, USA, 1705061) was used to visualize the target proteins. The membranes were stripped by boiling in PBS for 1 min, blocked again, and incubated with anti-β-actin primary antibody (Sigma-Aldrich, St. Louis, MO, USA, A5441) for 1 h at RT followed by an incubation with anti-mouse HRP secondary antibody (NEB 7076) for 1 h at RT.

### 2.17. Comparison of Proteome and Transcriptome Results

The UniProt identifiers of all proteins detected in the mass spectrometry analysis were mapped to the corresponding ensemble gene IDs using the UniProt Retrieve/ID mapping tool available at https://www.uniprot.org/id-mapping/ (accessed on 27 April 2022) with the following parameters: from database UniProtKB AC/ID to database Ensembl. Changes in gene/protein expression upon viral infections were compared using the UniProt/Ensembl identifiers and DESeq2/Proteus output data. The Splicify pipeline [25] was used for differential splice variant analysis in the transcriptome and proteome.

## 3. Results

### 3.1. Global Profiling of the Cellular Coding Transcriptome during Flavivirus Infections

**Figure 1:**
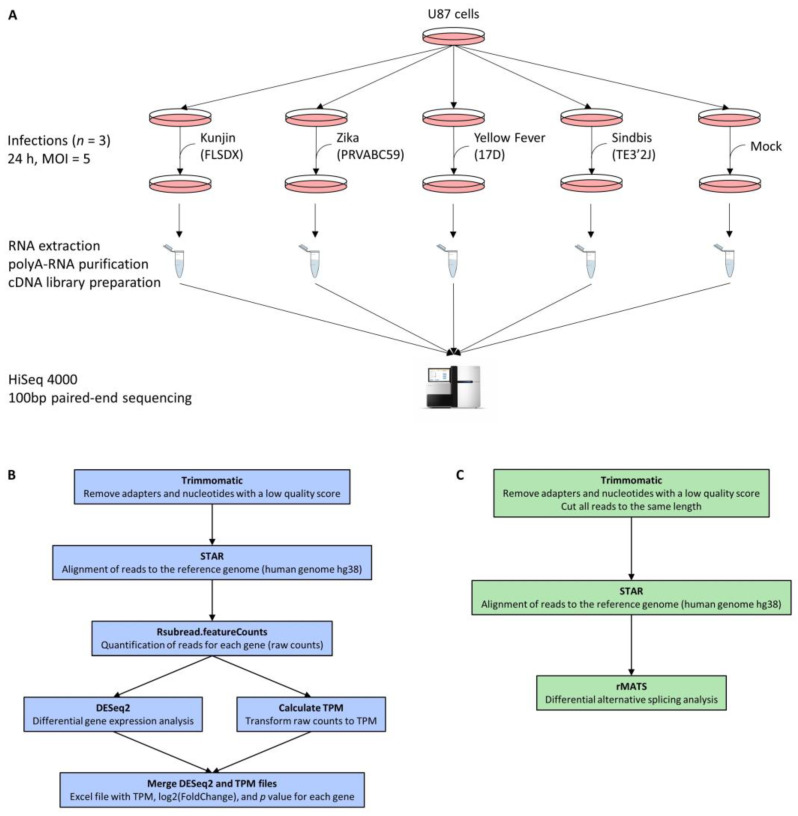
Experimental workflow. (**A**) The 50% confluent U87 cells were infected with either of the four viruses, or treated with cell-conditioned media, in biological triplicate. Then, 24 h post-infection, cells were harvested and RNA was isolated. PolyA-RNA was purified and used for the construction of cDNA libraries. Paired-end 100 bp sequencing was performed on a HiSeq 4000 system. (**B**,**C**) Data obtained from 100 bp paired-end RNA sequencing were analyzed for differential gene expression (**B**) and differential alternative splicing (**C**).

**Figure 2:**
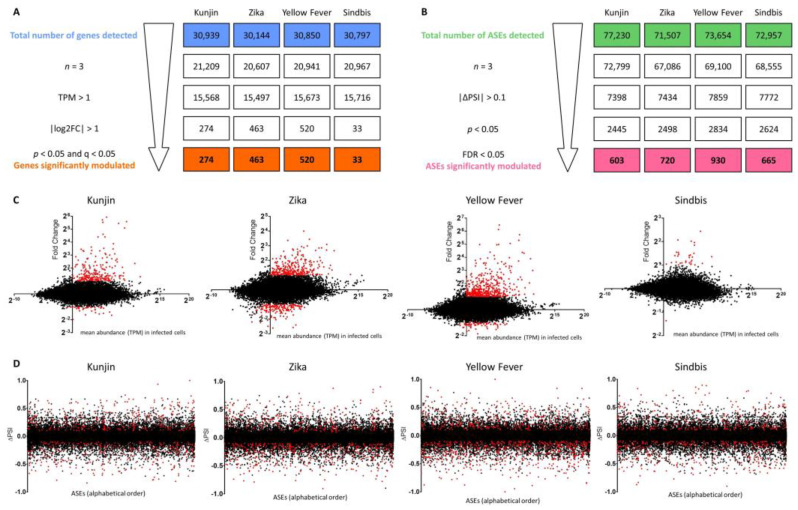
RNA-Seq data filtering. (**A**) Following differential gene expression analysis with DESeq2, the gene lists were filtered to retain only genes that were detected in triplicate in both experimental conditions (infected and non-infected cells), with an average abundance of more than 1 transcript per million (TPM) in at least one experimental condition, with an absolute log2 (fold-change) greater than 1, and p and q values below 0.05. (**B**) Following differential alternative splicing analysis with rMATS, the alternative splicing event (ASE) lists were filtered to retain only ASEs that were detected in triplicate in both the infected and non-infected cells, with an absolute ∆PSI greater than 0.1 (or 10%), and p and FDR values below 0.05. (**C**) The MA plots show fold changes between infected and non-infected cells (*y* axis) as well as gene abundance in infected cells (*x* axis) for each gene that was detected. Genes retained as being significantly up- or downregulated are displayed in red. (**D**) ∆PSI values for all detected alternative splicing events are displayed on the graphs, and ASEs that were significantly modulated upon infection are shown in red.

In this study, we investigated the changes in the coding transcriptome as well as in the proteome of virus-infected cells compared to non-infected cells, especially regarding differential gene expression and differential alternative splicing. We chose to include three closely-related Flaviviruses that cause distinct pathologies, as well as a fourth (+)ssRNA arbovirus from a different family (Sindbis virus, Alphavirus genus, Togaviridae family) to distinguish Flavivirus-specific effects on host cells from effects that are instead due to viral infection in general. Sindbis virus was chosen specifically since it is a positive-ssRNA mosquito-borne virus which can cause encephalitis, similar to some Flaviviruses. Given that two of the above-mentioned Flaviviruses are neurotropic, we chose to work with U87 cells, which were isolated from human gliomas, and are widely used [26]. U87 cells were infected at an MOI of 5 for 24 h with either KUNV, ZIKV, YFV, SINV, or treated with cell-conditioned media. PolyA-RNAs were purified prior to cDNA library construction and 100 nt paired-end RNA-Sequencing (Figure 1A). Viral infections were confirmed by ddPCR (Figure S1). At least 34 million reads were obtained for each sample (Figure S2). Reads were processed with Trimmomatic before being aligned to the reference genome (hg38 and viral genomes) using STAR. Rsubread.featureCounts was used to quantify reads for each gene prior to differential gene expression analysis using DESeq2, and rMATS was used for differential alternative splicing analysis (Figure 1B,C). Approximately 30,000 genes and 70,000 alternative splicing events (ASEs) were detected for each condition. The gene lists obtained from DESeq2 were filtered to retain only genes that were detected in triplicate in both experimental conditions (infected and non-infected cells), with an abundance of more than one transcript per million (TPM) in at least one experimental condition, with an absolute log2 (fold-change) greater than 1, and p and q values below 0.05. The ASE lists obtained from rMATS were filtered to retain only events that were detected in triplicate in both the infected and non-infected cells, with an absolute ∆PSI greater than 0.1 (or 10%), and p and FDR values below 0.05 (Figure 2). After applying these filters, several hundred genes were found to be significantly differentially expressed between virus-infected and mock-infected cells: 274 for KUNV, 463 for ZIKV, 520 for YFV, and 33 for SINV (Tables S1–S4). Moreover, several hundred ASEs were found to be significantly modulated during viral infections: 603 for KUNV, 720 for ZIKV, 930 for YFV, and 665 for SINV (Tables S5–S8).

### 3.2. Validation of RNA-Seq Data

**Figure 3:**
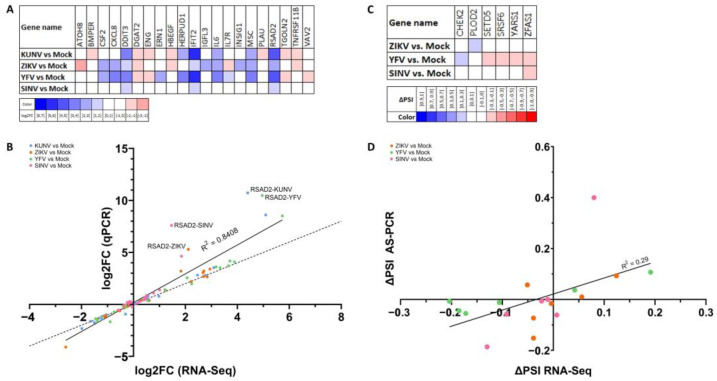
Validation of RNA-Seq data. (**A**) Overall, 21 genes were chosen for RT-qPCR validation of the RNA-Seq results. Their fold-changes in each infection compared to mock-infected cells are color-coded, with blue being upregulated, red being downregulated, and white meaning no significant change, according to the RNA-Seq data. (**B**) Fold-changes obtained from qPCR are plotted against fold-changes obtained from RNA-Seq. Both datasets correlate well (linear regression R^2^ = 0.84). Each dot represents one gene in one infection compared to control cells (blue: KUNV vs. Mock, orange: ZIKV vs. Mock, green: YFV vs. Mock, pink: SINV vs. Mock). The solid line is the linear regression, whereas the dashed line is the identity line (y = x). (**C**) Six alternative splicing events (ASEs) were chosen for the AS-PCR validation of the RNA-Seq results. Their ∆PSI values in each infection compared to mock-infected cells are color-coded, with blue favoring the short form in infected cells, red favoring the long form in infected cells, and white meaning no significant change, according to the RNA-Seq data. (**D**) ΔPSI values obtained from AS-PCR are plotted against ΔPSI values obtained from RNA-Seq. Each dot represents one ASE in one infection compared to control cells (orange: ZIKV vs. Mock, green: YFV vs. Mock, pink: SINV vs. Mock), and the solid line is the linear regression.

To validate the results obtained from RNA-Seq with a second approach, RT-qPCR was performed on 21 genes, the expression levels of which were found to be altered upon infection, as well as six ASEs that were dysregulated during infection (Figure 3, Table S9). Fold-changes as well as ∆PSI values obtained from both methods were correlated with R^2^ values of 0.84 (*p* < 0.0001) and 0.29 (*p* = 0.0193), respectively. This suggests that the results obtained from RNA-Seq are reliable, attaching significance to the subsequent analyses of the RNA-Seq results.

### 3.3. Alterations to Gene Expression Levels upon Viral Infection

**Figure 4:**
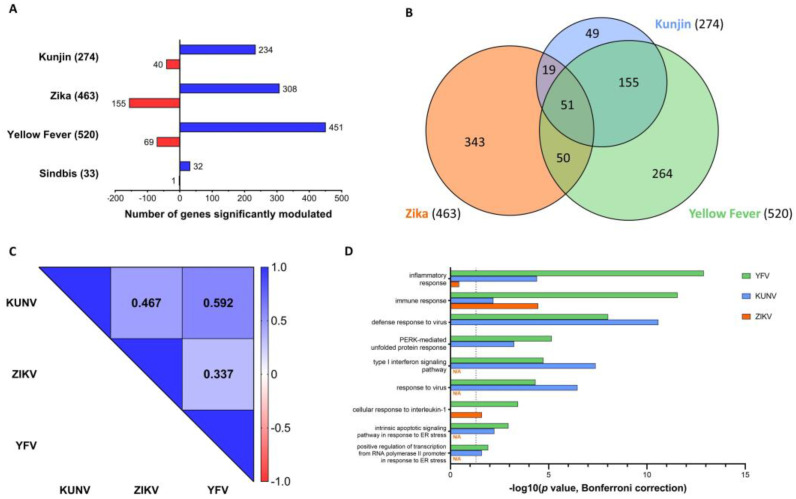
Genes with significant fold-changes upon viral infection. (**A**) The numbers of upregulated (blue) and downregulated (red) genes in each infection are shown. (**B**) The area-proportional Venn diagram shows overlaps in the sets of genes for which expression levels were significantly altered upon viral infection. (**C**) Genes that were detected in all experimental conditions and all replicates were used to calculate Spearman correlation coefficients. (**D**) Gene ontology enrichment analysis was performed, and GO terms that were significantly enriched (*p* value with Bonferroni correction < 0.05, represented by the dashed line) in at least two Flavivirus infections are shown. “N/A” indicates that no data was available.

We found that most cellular genes for which expression levels were modulated upon viral infections were overexpressed (Figure 4A). However, it should be noted that ZIKV repressed a higher proportion of differentially expressed genes (155/463 = 33%) than KUNV and YFV (40/274 = 15% and 69/520 = 13%, respectively). Considering that SINV only minimally affected host cell gene expression levels, it was not included in further analyses of the alterations to gene expression levels upon viral infection.

We next compared the genes with significant changes in each infection (Figure 4B) and found considerable overlaps between the three infections. Fisher’s Exact Tests yielded *p* values of 3.49 × 10^−54^ for KUNV/ZIKV, 1.32 × 10^−65^ for ZIKV/YFV, and 4.15 × 10^−288^ for KUNV/YFV. Interestingly, there is a greater overlap between the sets of genes for which expression levels were influenced by KUNV and YFV than between those influenced by KUNV and ZIKV or YFV and ZIKV, although KUNV and YFV genomes are phylogenetically more distant from each other than from ZIKV (Figure S3). Moreover, the majority (225/274 = 82%) of genes that were affected by KUNV were also dysregulated in at least one of the other infections, whereas most alterations in host gene expression levels upon infection with ZIKV (343/463 = 74%) and YFV (264/520 = 51%) were unique to those infections.

To confirm a higher correlation between changes to host gene expression levels induced by KUNV and YFV than by KUNV/ZIKV and YFV/ZIKV, Spearman correlation coefficients based on fold-change values of all genes detected in all experimental conditions (all three infections as well as mock-infected cells) were calculated (Figure 4C). The Spearman correlation coefficient for gene expression levels in KUNV/YFV-infected cells was 0.592 (*p* < 0.0001) compared to 0.467 (*p* < 0.0001) and 0.337 (*p* < 0.0001) for gene expression levels in KUNV/ZIKV- and YFV/ZIKV-infected cells, respectively. Scatter plots of fold-change values for all genes detected in all biological replicates in each experimental condition are shown in Figure S4. This suggests a higher similarity in the polyA-mRNA profile of cells infected with KUNV and YFV compared to cells infected with ZIKV.

Gene ontology enrichment analysis was performed on the genes for which expression levels were affected in each of the three Flavivirus infections, and the nine GO terms that were significantly enriched in at least two infections are shown in Figure 4D. Only the GO term ‘immune response’ was enriched in all three Flavivirus infections, whereas seven GO terms were enriched in both KUNV and YFV infections, and one GO term was enriched in ZIKV and YFV infections. This again indicates a higher similarity in the effects on the host cell by KUNV and YFV compared to ZIKV. Moreover, we compared the genes involved in the immune response that were affected in each infection (16 for KUNV, 25 for ZIKV and 35 for YFV), and found that only four genes were dysregulated in all three infections (Figure S5).

Interestingly, we found 17 genes associated with the GO term ‘nervous system development’ (DCLK1, FGF2, INHBA, CXCL1, DOK5, TPP1, ENC1, BDNF, CRIM1, MYLIP, JAG1, NRG1, NDP, PCDH18, FOS, DLX5, ATOH8) to be differentially expressed upon infection with ZIKV, although this GO term was not significantly enriched (Bonferroni-corrected *p* = 0.36). Only 4 of these 17 genes were found to be differentially expressed upon infection with YFV, and none of them were associated with altered gene expression levels in KUNV-infected cells (Table S10). This correlates with the fact that ZIKV is the only Flavivirus known to affect brain development and cause microcephaly [27], and it would be interesting to study the role of these genes and their expression levels in this regard.

### 3.4. Modulations Regarding Alternative Splicing Events (ASEs) during Viral Infection

**Figure 5:**
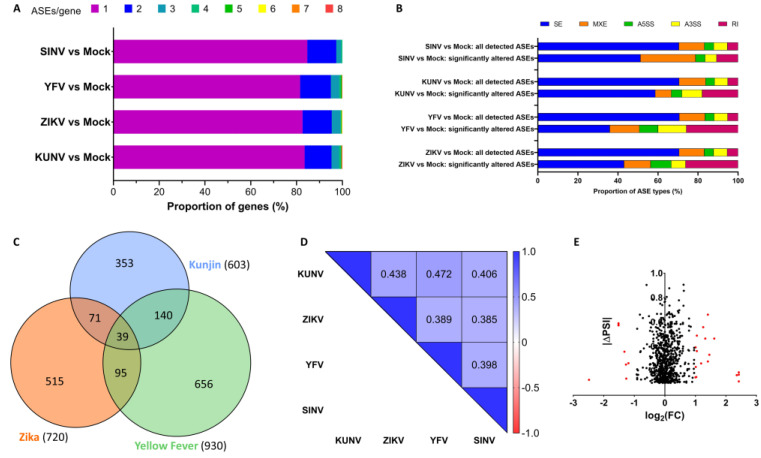
ASEs modulated during viral infection. (**A**) For all genes that had at least one ASE significantly altered upon viral infection, the number of ASEs per gene is shown. (**B**) The proportions of the different types of ASEs (SE: skipped exon, MXE: mutually exclusive exons, A5SS: alternative 5′ splice site, A3SS: alternative 3′ splice site, RI: retained intron) among all events analyzed as well as the events that were significantly changed upon viral infections are shown. (**C**) The area-proportional Venn diagram shows overlaps in the sets of ASEs that were significantly altered during viral infections. (**D**) ASEs that were detected in all experimental conditions and all replicates were used to calculate Spearman correlation coefficients. (**E**) The fold-change values for all genes that had at least one ASE significantly altered upon ZIKV infection are shown. Each dot represents one significantly modulated ASE, and red dots represent events on genes that are also overexpressed or repressed.

One pre-mRNA can contain many splice sites that may be differentially regulated during viral infection [28]. We therefore investigated the number of significantly altered ASEs per gene in virus-infected cells compared to mock-infected cells. In more than 80% of the genes in which AS was modulated upon infection, only one ASE was altered, and very few genes were subject to the dysregulation of more than three ASEs (Figure 5A). We also analyzed the nature of the ASEs which were modulated upon infection and found an underrepresentation of skipped exons and an overrepresentation of retained introns among the ASEs dysregulated in viral infections compared to all ASEs analyzed (Figure 5B).

When comparing the ASEs dysregulated in each infection, there are significant overlaps between ASEs changed in the three Flavivirus infections, with Exact Fisher’s Test *p* values of 1.65 × 10^−87^, 9.25 × 10^−92^ and 3.64 × 10^−166^ for KUNV/ZIKV, ZIKV/YFV, and KUNV/YFV, respectively (Figure 5C). Nevertheless, more than half of the ASEs that were significantly modulated by each virus (353/603 = 59% for KUNV, 515/720 = 72% for ZIKV, 656/930 = 71% for YFV) were found to be unique to that particular infection. Spearman correlation coefficients based on ∆PSI values of all ASEs detected in all experimental conditions (all infections as well as mock-infected cells) were calculated and are shown in Figure 5D. Interestingly, correlation coefficients obtained when comparing Flavivirus infections with each other and Flavivirus infections with the SINV infection are very similar. Taken together, these results suggest that there are many more dysregulated ASEs common to infections than one would expect randomly, indicating that viruses have rather similar effects on alternative splicing. However, infections with closely related viruses do not share significantly more altered ASEs than infections with viruses that have a greater phylogenetic distance.

Gene ontology enrichment analysis was performed with the genes that were subject to modulations in alternative splicing. However, no biological process was significantly enriched. Nonetheless, we found several significantly modulated ASEs that might be interesting to characterize further in future studies. For example, regarding genes involved in the immune response, an increase in the retention of intron 12 in the STAT2 pre-mRNA as well as an increase in the retention of intron 7 in the IRF7 pre-mRNA were observed in ZIKV and YFV infections (ΔPSI of −0.111/−0.165 and −0.336/−0.598, respectively). In both cases, the intron retention leads to the occurrence of a premature termination codon in the mRNA (Figure S7).

Finally, we compared the genes in which expression levels were altered, and the genes in which alternative splicing was affected upon viral infection. We found that only very few genes that were subjected to the dysregulation of one or more ASEs were also overexpressed or repressed (Figure 5E and Figure S6). Only one gene, PNLIPRP3, showed dysregulated expression levels (overexpression) as well as dysregulated alternative splicing in all three Flavivirus infections (the same three ASEs in KUNV and ZIKV infection, but only one of the three ASEs in YFV infection). Moreover, the protein–protein interaction network and functional enrichment analysis with STRING revealed, among other things, an enrichment for genes involved in the ER unfolded protein response, among the genes in which expression levels, as well as alternative splicing, were significantly modulated upon infection with KUNV and YFV (FDR of 0.0025 and 0.0100, respectively). However, no biological process was enriched in the case of ZIKV infection (Figure S6).

### 3.5. Changes in the Host Proteome upon Flavivirus Infections

**Figure 6:**
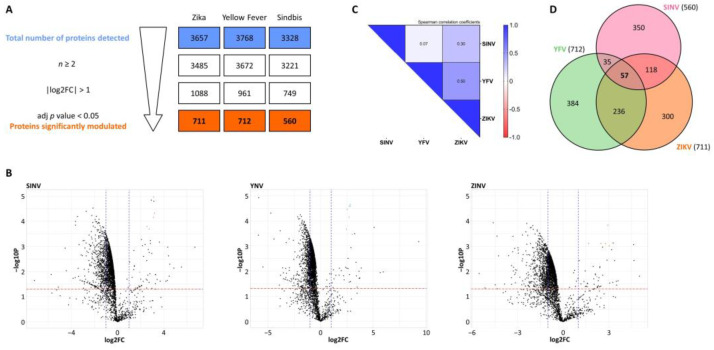
Host cell proteome upon viral infections. (**A**) Following differential protein expression analysis with Proteus, the protein lists were filtered to retain only proteins that were detected in at least two replicates, with an absolute log2 (fold-change) greater than 1, and an adjusted *p* value below 0.05. (**B**) Volcano plots show fold changes in protein expression. Black dots represent host proteins, whereas orange/green/pink dots represent viral (ZIKV/YFV/SINV) proteins. (**C**) Log2FC values of proteins that were detected in at least two infections were used to calculate Spearman correlation coefficients. (**D**) The area-proportional Venn diagram shows overlaps in the sets of proteins for which expression levels were significantly altered during viral infections.

Given the considerable number of changes observed in the coding transcriptome of the host cells, both in terms of gene expression and alternative splicing, we wondered if they affected only the RNA level, or if they were translated into modifications in the host cell proteome. We used SILAC to detect differences in protein abundance among infected and non-infected cells. Briefly, U87 cells were cultured in the presence of light, medium or heavy L-arginine (R0/R6/R10), and L-Lysine (K0/K4/K8) before being treated with cell-conditioned media (R0K0) or infected with either ZIKV (R6K4), YFV (R6K4), or SINV (R10K8). Equal amounts of total protein from light, medium, and heavy lysates were mixed, proteins were digested using trypsin, and peptides were analyzed using mass spectrometry. MaxQuant (Max Planck Institute of Biochemistry, Martinsried, Germany) was used to identify and quantify peptides, and differential protein expression analysis was performed with Proteus. Approximately 3500 proteins were detected for each condition. The protein lists obtained from Proteus were filtered to retain only proteins that were detected in at least two replicates, with an absolute log2 (fold-change) greater than 1, and an adjusted *p* value below 0.05 (Figure 6A). After applying these filters, several hundred proteins were found to be significantly differentially expressed between virus-infected and mock-infected cells: 711 for ZIKV, 712 for YFV, and 560 for SINV (Tables S11–S13). We found that most proteins were repressed upon viral infection (Figure 6B). Only a few dozen proteins were overexpressed in infected cells, including viral proteins.

Fold-change values of all proteins detected in at least two infections were used to calculate Spearman correlation coefficients (Figure 6C). The proteomes of cells infected with ZIKV and YFV were more alike than cells infected with SINV, with correlation coefficients of 0.50 for ZIKV/YFV (*p* = 3.86 × 10^−169^), 0.30 for ZIKV/SINV (*p* = 5.24 × 10^−62^), and 0.007 for YFV/SINV (*p* = 0.0004). The scatter plots of fold-change values for all proteins detected in at least two infections are shown in Figure S8. Moreover, the lists of proteins significantly differentially expressed in each infection were compared (Figure 6D), and overlaps were found to be significant (*p* values of 1.29 × 10^−23^ for ZIKV/YFV, 1.04 × 10^−4^ for ZIKV/SINV, and 3.19 × 10^−13^ for YFV/SINV). A greater overlap between differentially expressed proteins in Flavivirus infections was observed compared to the infection with SINV, and 57 proteins were found to be affected in all three infections.

**Figure 7:**
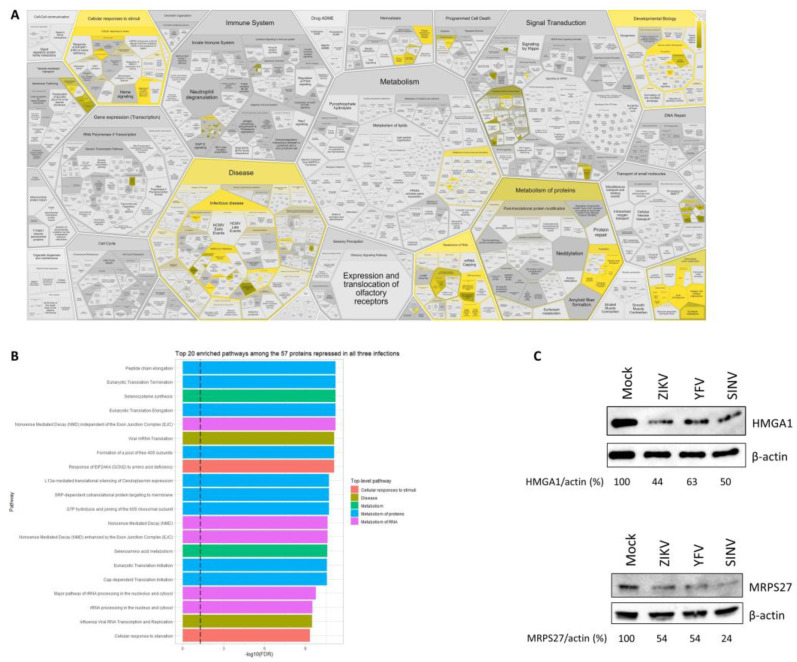
Functions of the 57 proteins repressed in all three infections. (**A**) Reactome over-representation analysis results. (**B**) The top 20 enriched pathways and their associated -log10 (FDR) are shown. (**C**) Western blot analysis confirms the downregulation of HMGA1 (involved in the regulation of gene transcription) and MRPS27 (involved in protein synthesis) in infected cells.

All 57 proteins that were common to the three infections were repressed upon infection, and Reactome overrepresentation analysis revealed an enrichment for pathways involved in the cellular response to stimuli (stress), (infectious) disease, the metabolism of RNA, metabolism of proteins, developmental biology, and extracellular matrix organization (Figure 7).

### 3.6. Validation of SILAC Data

We next selected two of the most downregulated proteins in Flavivirus infections (previously identified in the SILAC experiment), HMGA1 and MRPS27, for validation by Western blot. HMGA1 is associated with the cellular response to stimuli as well as disease, whereas MRPS27 plays a role in the metabolism of proteins. As shown in Figure 7C, Western blot analysis shows a downregulation of both proteins in infected cells, which correlates with the changes in protein expression found through mass spectrometry analysis.

### 3.7. Comparison of Proteome and Transcriptome Results

**Figure 8:**
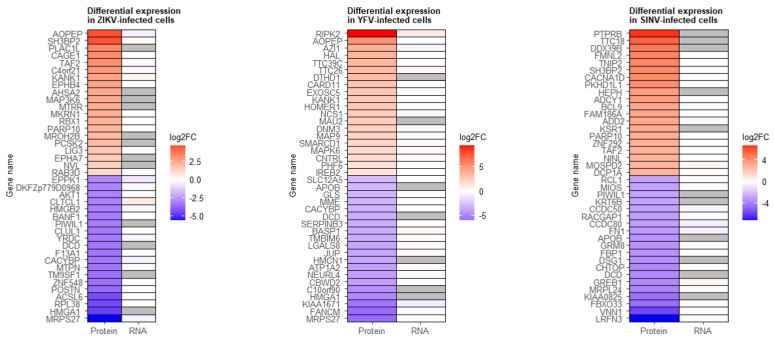
Comparison of proteome and transcriptome results. The top 20 overexpressed and repressed proteins in each infection, along with the fold-change values of their mRNAs, are shown. Red represents positive fold-change values (overexpression in infected cells), blue represents negative fold-change values (repression in infected cells), and gray represents missing data. Only host proteins were included in this analysis, and viral proteins were excluded.

As previously mentioned, the majority of genes in which expression was affected in the viral infections were overexpressed (Figure 4A), whereas most proteins with significant changes in expression upon infection were downregulated (Figure 6B). To further investigate any correlation or disconnect in alterations in gene/protein expression in infected cells, we looked at the top 20 overexpressed and repressed proteins in each infection and their corresponding mRNAs. As shown in Figure 8, the expression levels of these mRNAs are minimally affected. It is noteworthy that for several proteins, no data are available regarding their mRNAs. For a more global comparison of proteome and transcriptome results, we examined all genes/proteins for which data were available in both the RNA-Seq and SILAC experiments. Figure S9 shows scatter plots of fold-changes in protein and RNA expression. Taken together, these results suggest the absence of correlation between mRNA and protein expression levels following viral infections.

We also investigated whether the changes in alternative splicing observed in the RNA-Seq experiment had an impact on protein isoforms. The proteogenomic pipeline Splicify [25] was applied to our RNA-Seq and mass spectrometry data to identify differentially expressed splice variants. Despite many significant changes in alternative splicing observed in the RNA-Seq data described above, no significantly differentially expressed peptides corresponding to these alternative splicing events were found. This further suggests a disconnect between the transcriptome and the proteome of virus-infected cells.

## 4. Discussion

In the present study, we report the effects of three Flaviviruses, namely KUNV, ZIKV, and YFV, on gene expression levels and alternative splicing in U87 cells. Previous RNA-Seq studies mostly focused on a single viral species, sometimes comparing different strains of the same species, and most of them examined either gene expression levels or alternative splicing [11,12,13,14,15]. Here, we present our findings regarding the modulations of gene expression levels as well as alternative splicing upon infection with three different Flavivirus species under the same experimental conditions, allowing for comparisons between KUNV, ZIKV, and YFV, and their effects on the coding transcriptome of their host cells. Moreover, we examined the proteome of cells infected with these viruses to determine whether modulations in the coding transcriptome observed via RNA-Seq translated into alterations in the proteome.

Upon infection with KUNV, ZIKV, and YFV, we observed 274, 463, and 520 genes to be differentially expressed compared to non-infected cells. These numbers are lower compared to the number of genes/transcripts that have been identified in other RNA-Seq studies with Flavivirus-infected cells [12,15], possibly due to different experimental conditions and/or bioinformatic tools used. Moreover, we identified only 33 genes to be differentially expressed upon infection with SINV. Given that SINV replicates faster and is more cytopathic than Flaviviruses [29], it might kill cells faster and not allow for many significant changes in gene expression levels. In the three Flavivirus infections, most of the differentially expressed genes were found to be overexpressed. This could be due to the cell activating the expression of genes that produce proteins with antiviral functions [1]. It could also be an apparent upregulation as a result of increased mRNA stability. In fact, subgenomic flavivirus RNA (sfRNA), which is derived from the 3′ UTR of the viral genome, has been shown to sequester and inactivate the exonuclease XRN1, thus increasing the stability of cellular mRNAs [30,31].

Gene ontology enrichment analysis revealed 17 genes associated with the GO term ‘nervous system development’, the expression of which was dysregulated upon infection with ZIKV. These genes include BDNF and NRG1, which are involved in neuronal survival and growth [32,33] and are both repressed in ZIKV-infected cells. More specifically, NRG1 has been shown to be expressed in the early stages of brain development [33], and it would be interesting to investigate the effect of its repression with respect to ZIKV-induced microcephaly [34]. In addition, since decreased levels of BDNF have been observed in several neurodegenerative diseases [32], and ZIKV is potentially associated with the development and/or progression of neurodegenerative diseases [35], it would be interesting to study the role of BDNF repression in this context. Two other attractive candidates for further studies are the genes DLX5 and ATOH8, which are also repressed in ZIKV-infected cells. DLX5 has been shown to be involved in the development of the olfactory system [36], and ZIKV has recently been associated with olfactory disorders in mice [37] as well as reduced olfactory function in patients with ZIKV-induced Guillain-Barré syndrome [38]. ATOH8 is involved in the developing nervous system as well as the inner ear [39], and ZIKV has been observed to cause hearing loss [40]. Further studies on the roles of the downregulation of these genes may shed more light on ZIKV pathogenesis.

In addition to altered gene expression levels, RNA-Seq revealed modulations in several hundred alternative splicing events upon viral infection: 603 for KUNV, 720 for ZIKV, 930 for YFV, and 665 for SINV. In contrast to what was observed for gene expression levels, changes in alternative splicing induced by the three Flaviviruses and by SINV are of the same order of magnitude. Moreover, when comparing the effects of the different viruses on host cell alternative splicing, no greater similarity among Flavivirus infections compared to the infection with SINV was observed. This suggests that alterations in cellular alternative splicing are caused by viral infections in general, rather than being Flavivirus-specific.

To shed light on the mechanism behind the alterations in alternative splicing, we further investigated 212 known splicing factors. None of their mRNAs were found to be differentially expressed upon infection (Table S14). Therefore, the observed changes in host cell alternative splicing are most likely not due to an up- or downregulation of splicing factors. Interestingly, we found several alternative splicing events on the mRNAs of splicing factors to be significantly modulated upon viral infection (Table S15). These modulations could lead to the degradation of the mRNA or the translation of different isoforms of these splicing factors, and further studies are needed to examine the functional consequences of these alterations in alternative splicing.

Upon the identification of several hundred modulations in host cell gene expression levels and alternative splicing events via RNA sequencing, we wondered if these changes in the transcriptome could result in changes at the protein level. We analyzed the proteome of virus-infected cells for alterations in protein abundance and isoforms using SILAC. The differential expression of several hundred proteins (711 for ZIKV, 712 for YFV, and 560 for SINV) was observed, and the majority of these proteins were found to be repressed upon infection. This is somewhat surprising, given that the RNA-Seq revealed that most genes that were affected by the viruses were upregulated. However, many viruses are known to induce a translation shutoff of cellular genes to promote the translation of viral proteins [29,41,42], which could explain our observations. Moreover, the analysis of our data with the proteogenomic pipeline Splicify [25] did not identify any significant differential splice variants within the proteome of infected cells. Further studies are needed to better understand the impact of transcriptomic modulations, which occur upon infection, on the functions of the host cell.

The observation that most differentially expressed transcripts were upregulated whereas most differentially expressed proteins were downregulated in infected cells, in addition to the detection of several hundred differentially regulated ASEs in the transcriptome but no matching differential splice variants in the proteome, suggests a disconnect between the transcriptome and the proteome in Flavivirus-infected cells. We therefore analyzed the fold-changes of the differentially expressed proteins and the fold-changes of the corresponding mRNAs and found no significant correlation. One possible reason for the disconnect between mRNA regulation and protein expression could be that the viruses use the mRNAs themselves to control viral or host cell activities, as is the case with sfRNA, rather than dysregulating mRNA levels to affect protein abundance. The lack of correlation between mRNA and protein levels could also be due to post-transcriptional and post-translational regulation. For example, the stability of mRNAs could be increased due to the sequestration of XRN1 by sfRNA, whereas the half-life of the corresponding protein is unaffected [30,31]. Alternatively, proteins may be ubiquitinated and degraded [43] while mRNA levels are unchanged. A third option would be the repression of translation and sequestering of mRNAs in stress granules [44], leading to low protein abundance despite high amounts of mRNAs. Overall, our findings are in agreement with previous studies on DENV- and JEV-infected cells that have reported little or no overlap between differentially expressed transcripts and protein levels [45,46].

In this study, we report a global view of the coding transcriptome and the proteome of virus-infected cells compared to non-infected cells, but little is known about the mechanisms behind the modulations that are observed for mRNA and protein abundance as well as mRNA alternative splicing. These alterations could be brought about directly by the virus, initiated by the host cell as a defense mechanism, or be connected to the antiviral response. On the one hand, the viruses could induce these changes for their benefit, for example, to promote viral replication or to inhibit the immune response. In fact, DENV NS5 has been shown to interact with the spliceosome and modulate the splicing of cellular transcripts [47]. Moreover, DENV NS5 has been reported to trigger the degradation of the splicing factor RBM10, leading to the alternative splicing of the SAT1 pre-mRNA. This prompts the degradation of the transcript and a decrease in the functional SAT1 protein with antiviral activity [16]. On the other hand, the host cell could induce these changes to fight the infection and limit viral replication. For example, upon the detection of viral components, the host cell produces interferons and induces the expression of many interferon-stimulated genes, which play a role in the antiviral response [1]. Most likely, the observed modulations are a combination of virus-induced and host-cell-induced alterations, and further studies will be needed to elucidate the role of each of them, as well as the mechanisms behind them.

## Data Availability

RNA-Seq data have been deposited with the Gene Expression Omnibus (GEO) [48] under accession number GSE232504. Mass spectrometry proteomics data have been deposited to the ProteomeXchange Consortium [49] via the proteomics identification database (PRIDE) partner repository [50] with the dataset identifier PXD042208.

## Supplementary Materials

The following supporting information can be downloaded at https://www.mdpi.com/article/10.3390/v15071419/s1. Figure S1. Quantification of viral RNA. Abundance of viral RNAs in total RNA extracts from infected cells was measured using droplet digital PCR (ddPCR). To correct for differences in the amounts of input cDNA, four housekeeping genes (B2M, MRPL19, PUM1, YWHAZ) were quantified, and copies/μL of viral cDNA were divided by copies/μL of housekeeping genes. Figure S2. RNA-Seq data. PolyA-enriched cDNA libraries from infected (Kunjin, Zika, Yellow Fever, Sindbis) and non-infected (control) cells were subjected to 100 bp paired-end sequencing using the Illumina HiSeq4000 system. At least 34 million reads were obtained for each sample. At least 83% of the reads were uniquely mapped to the reference genome. Figure S3. Flavivirus phylogenetic tree. The polyprotein sequences of the three Flaviviruses used in this study were aligned using SeaView5/Clustal Omega, and a phylogenetic tree was constructed based on this alignment using SeaView5/PhyML. Figure S4. Correlations in changes to gene expression levels and alternative splicing upon viral infection. (A) Fold-change values (log2) for all genes detected in every experimental condition are shown. (B) ΔPSI values for all ASEs detected in every experimental condition are shown. Figure S5. Gene ontology enrichment analysis results for the term ‘immune response’. The GO term ‘immune response’ was found to be significantly enriched among the genes in which expression levels were altered following all three Flavivirus infections. Here, the genes involved in the immune response that are affected in each infection are shown. Figure S6. Expression levels of genes with one or more dysregulated ASEs upon infection. (A) The absolute ∆PSI values as well as fold-change values for all genes with at least one dysregulated ASE are shown. Each dot represents one significantly modulated ASE, and red dots represent events on genes that are also significantly overexpressed or repressed. (B) The area-proportional Venn diagram shows overlaps in the sets of genes for which expression levels as well as alternative splicing were significantly altered upon each viral infection. (C) An analysis of the genes that showed dysregulated expression levels as well as alternative splicing was performed with STRING. One of the most significantly enriched biological processes in KUNV and YFV infections was ‘GO:0030968 endoplasmic reticulum unfolded protein response’ (FDR = 0.0025 and 0.0100, respectively), and the proteins involved in these processes are highlighted with black circles. No biological process was significantly enriched among the genes dysregulated in the ZIKV infections. Figure S7. Intron retention in genes involved in the immune response. (A) Increased retention of intron 12 in the STAT2 mRNA in infected cells is shown. (B) Increased retention of intron 7 in the IRF7 mRNA is shown. Figure S8. Correlations in changes to protein expression levels upon viral infection. Scatter plots of fold-change values for all proteins detected in at least two infections are shown. Figure S9. Comparison of transcriptome and proteome results. (A) Scatter plots of fold-change values for all proteins and their mRNAs detected in each viral infection are shown. (B) Spearman correlation coefficients of RNA and protein fold-changes in each infection, along with their *p* values, are displayed. Table S1. Changes in gene expression levels upon infection with Kunjin virus. Overall, 274 genes were found to be differentially expressed between KUNV-infected and mock-infected U87 cells. Table S2. Changes in gene expression levels upon infection with Zika virus. In total, 463 genes were found to be differentially expressed between ZIKV-infected and mock-infected U87 cells. Table S3. Changes in gene expression levels upon infection with Yellow Fever virus. In total, 520 genes were found to be differentially expressed between YFV-infected and mock-infected U87 cells. Table S4. Changes in gene expression levels upon infection with Sindbis virus. In total, 33 genes were found to be differentially expressed between SINV-infected and mock-infected U87 cells. Table S5. Changes in alternative splicing upon infection with Kunjin virus. In total, 603 alternative splicing events were found to be significantly different between KUNV-infected and mock-infected U87 cells. Overall, there were (A) 61 alternative 3’ splice sites; (B) 32 alternative 5’ splice sites; (C) 48 mutually exclusive exons; (D) 108 retained introns; (E) 354 skipped exons. Table S6. Changes in alternative splicing upon infection with Zika virus. In total, 720 alternative splicing events were found to be significantly different between ZIKV-infected and mock-infected U87 cells: (A) 43 alternative 3’ splice sites; (B) 62 alternative 5’ splice sites; (C) 80 mutually exclusive exons; (D) 158 retained introns; (E) 377 skipped exons. Table S7. Changes in alternative splicing upon infection with Yellow Fever virus. In total, 930 alternative splicing events were found to be significantly different between YFV-infected and mock-infected U87 cells: (A) 85 alternative 3’ splice sites; (B) 56 alternative 5’ splice sites; (C) 89 mutually exclusive exons; (D) 156 retained introns; (E) 544 skipped exons. Table S8. Changes in alternative splicing upon infection with Sindbis virus. In total, 665 alternative splicing events were found to be significantly different between SINV-infected and mock-infected U87 cells: (A) 38 alternative 3’ splice sites; (B) 32 alternative 5’ splice sites; (C) 183 mutually exclusive exons; (D) 71 retained introns; (E) 341 skipped exons. Table S9. Validation of changes in gene expression levels. In total, 21 genes were chosen for qRT-PCR validation of the RNA-Seq results. Table S10. Expression levels of genes associated with the GO term ‘nervous system development’. Genes significantly overexpressed are shown in blue, and genes significantly repressed are shown in red. Table S11. Changes in protein expression levels upon infection with Zika virus. In total, 711 proteins were found to be differentially expressed between ZIKV-infected and mock-infected U87 cells. Table S12. Changes in protein expression levels upon infection with Yellow Fever virus. In total, 712 proteins were found to be differentially expressed between YFV-infected and mock-infected U87 cells. Table S13. Changes in protein expression levels upon infection with Sindbis virus. In total, 560 proteins were found to be differentially expressed between SINV-infected and mock-infected U87 cells. Table S14. Gene expression levels of known splicing factors. No known splicing factor was found to be significantly differentially expressed upon infection with KUNV, ZIKV, YFV, or SINV. Table S15. Alternative splicing of known splicing factors. Several alternative splicing events on mRNAs encoding known splicing factors were found to be significantly altered upon infection with KUNV, ZIKV, YFV, or SINV.
