# Supplementary figures and images for: Kunjin Virus, Zika Virus, and Yellow Fever Virus Infections Have Distinct Effects on the Coding Transcriptome and Proteome of Brain-Derived U87 Cells
Source: Viruses. 2023 Jun 23;15(7):1419. doi: 10.3390/v15071419 (PMC10385720; doi:10.3390/v15071419)

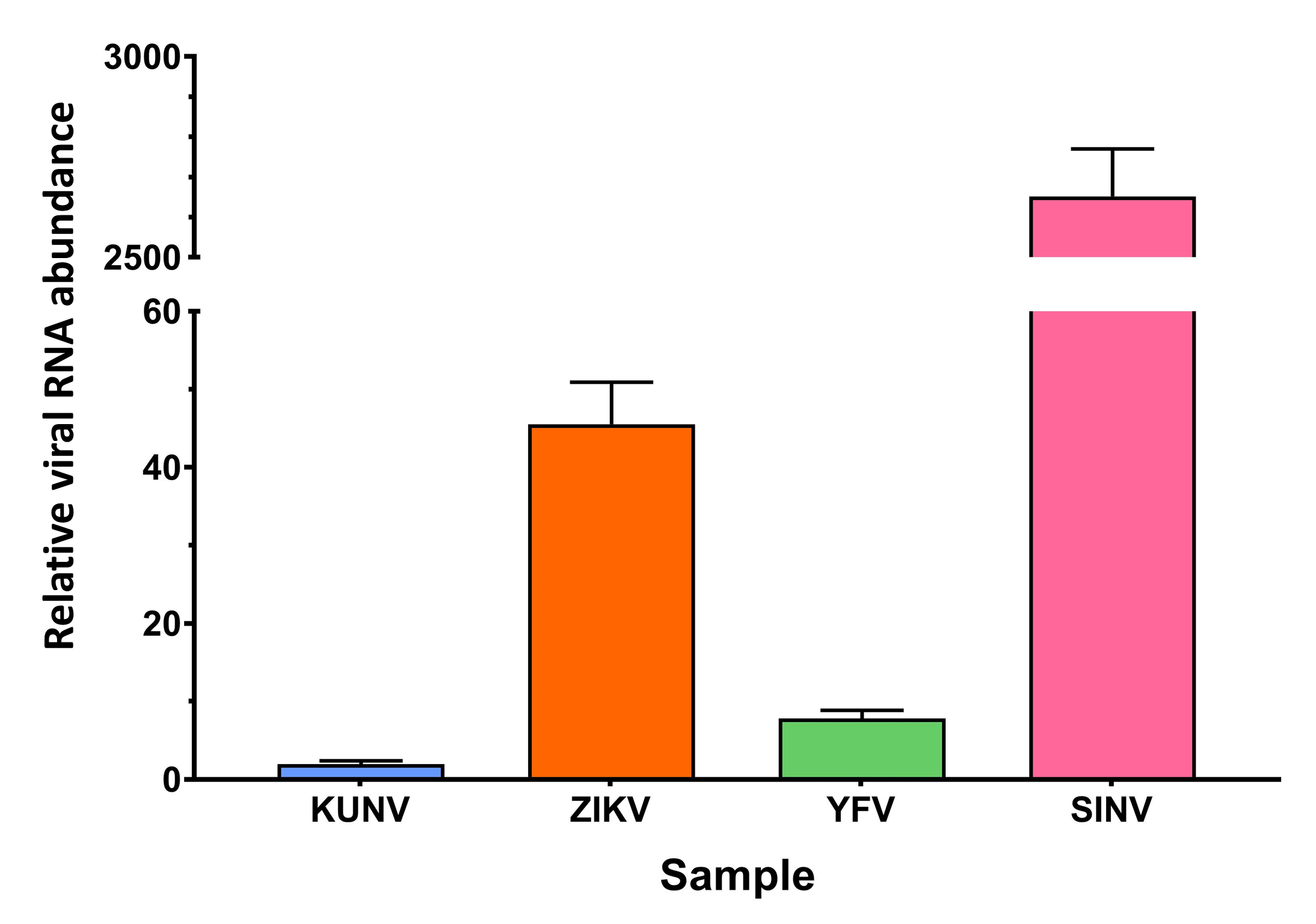

Supplement: Supplementary file 1 [file viruses-15-01419-s001.zip › FigureS1.TIF]

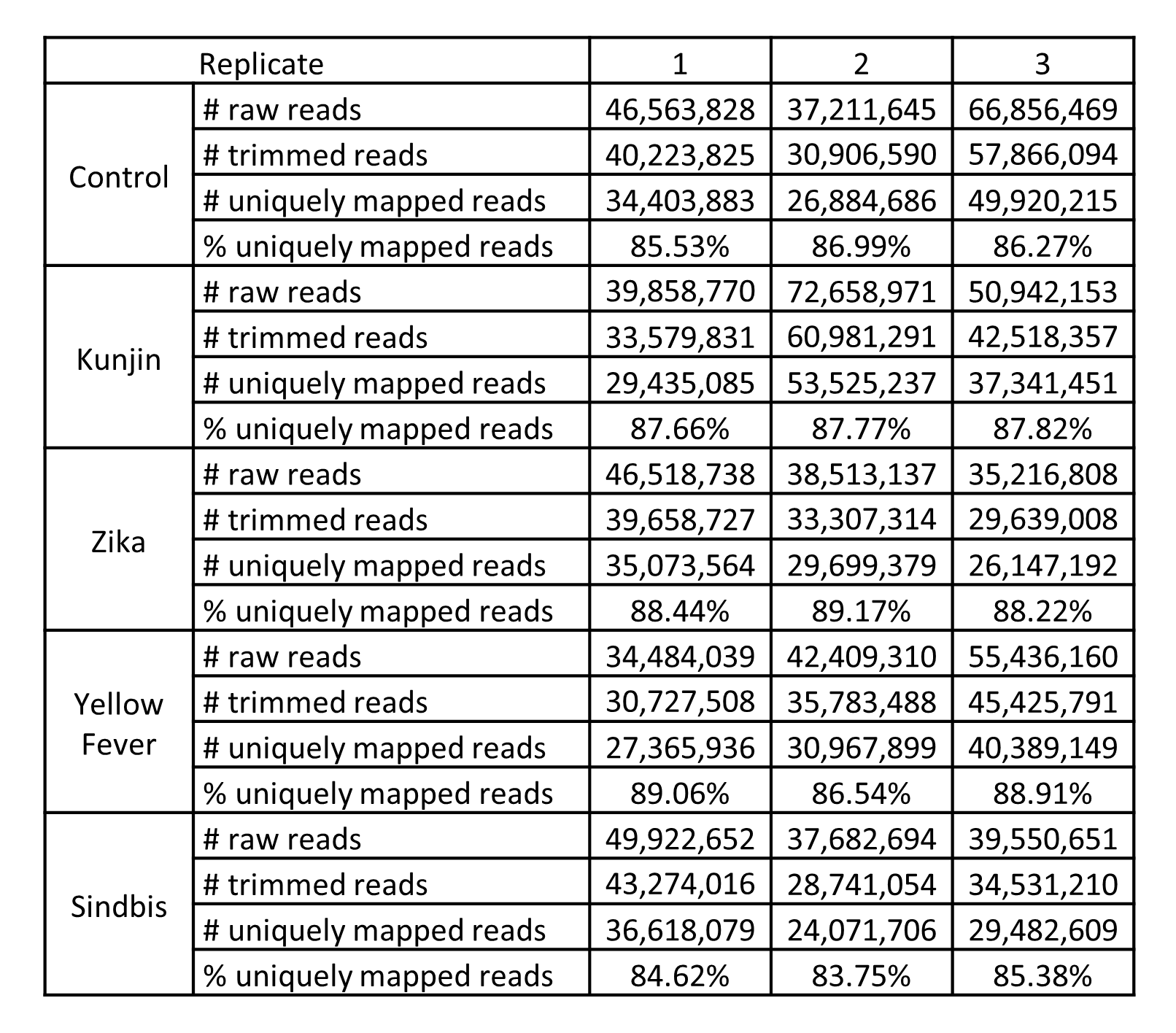

Supplement: Supplementary file 1 [file viruses-15-01419-s001.zip › FigureS2.TIF]

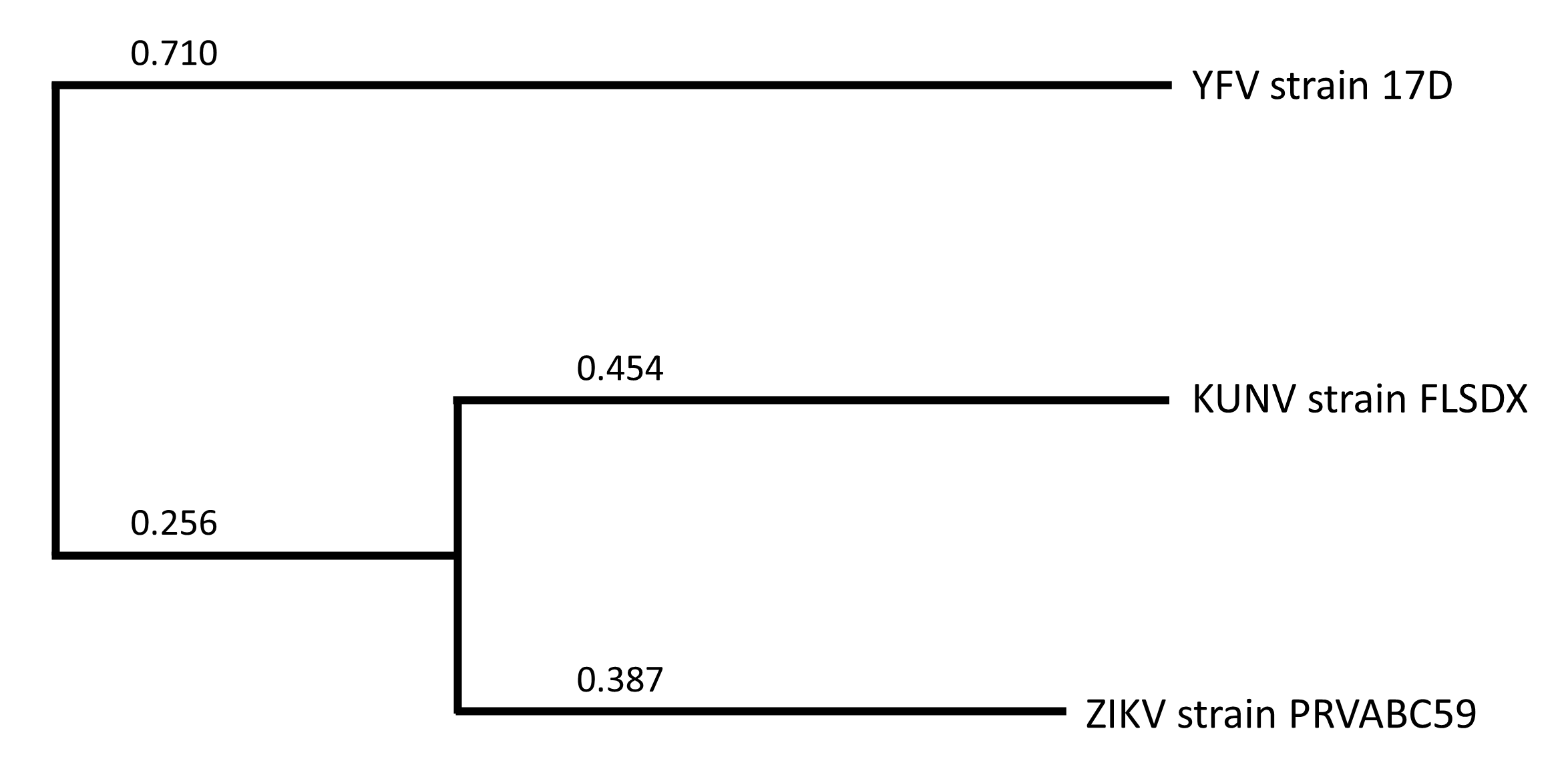

Supplement: Supplementary file 1 [file viruses-15-01419-s001.zip › FigureS3.TIF]

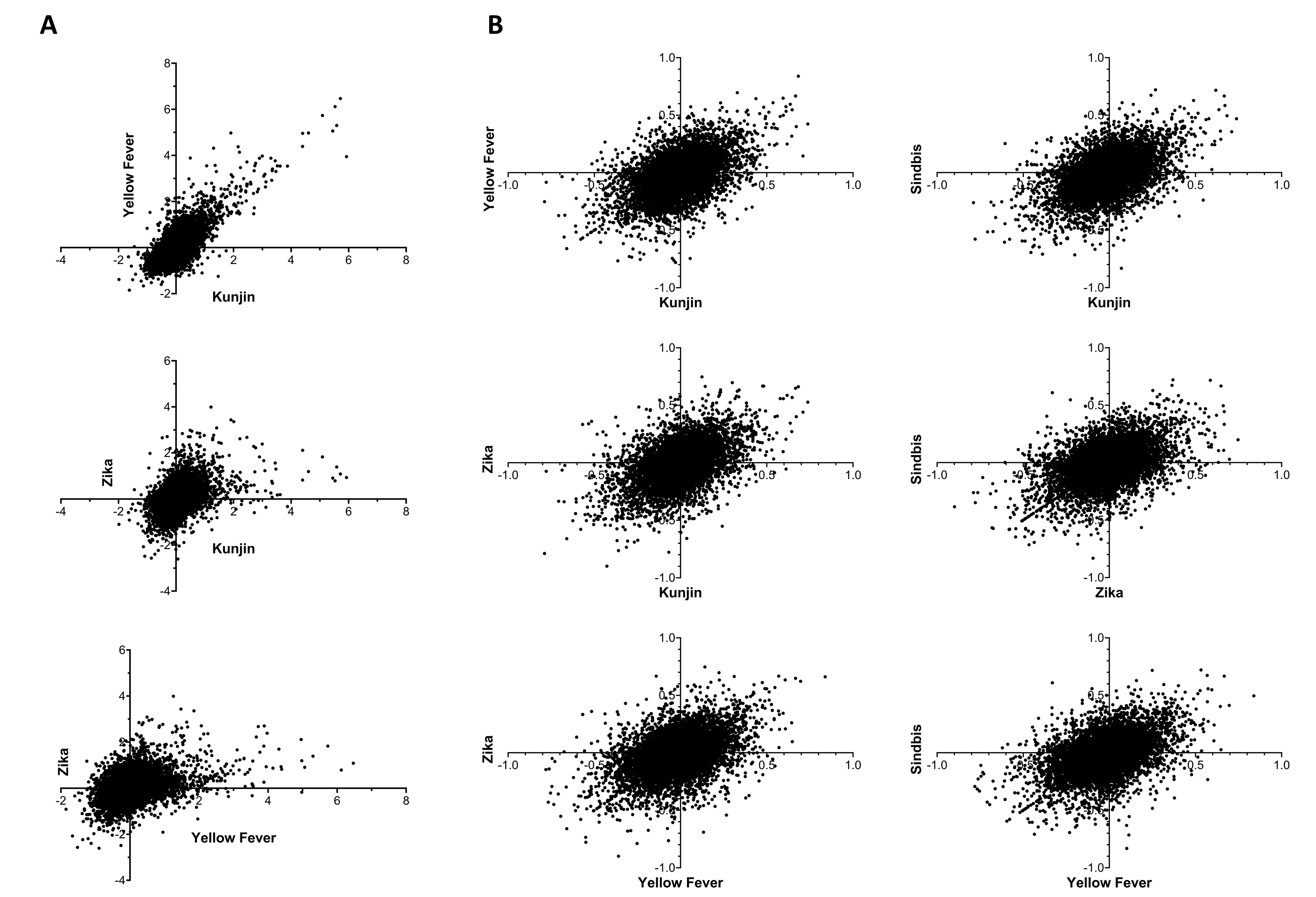

Supplement: Supplementary file 1 [file viruses-15-01419-s001.zip › FigureS4.TIF]

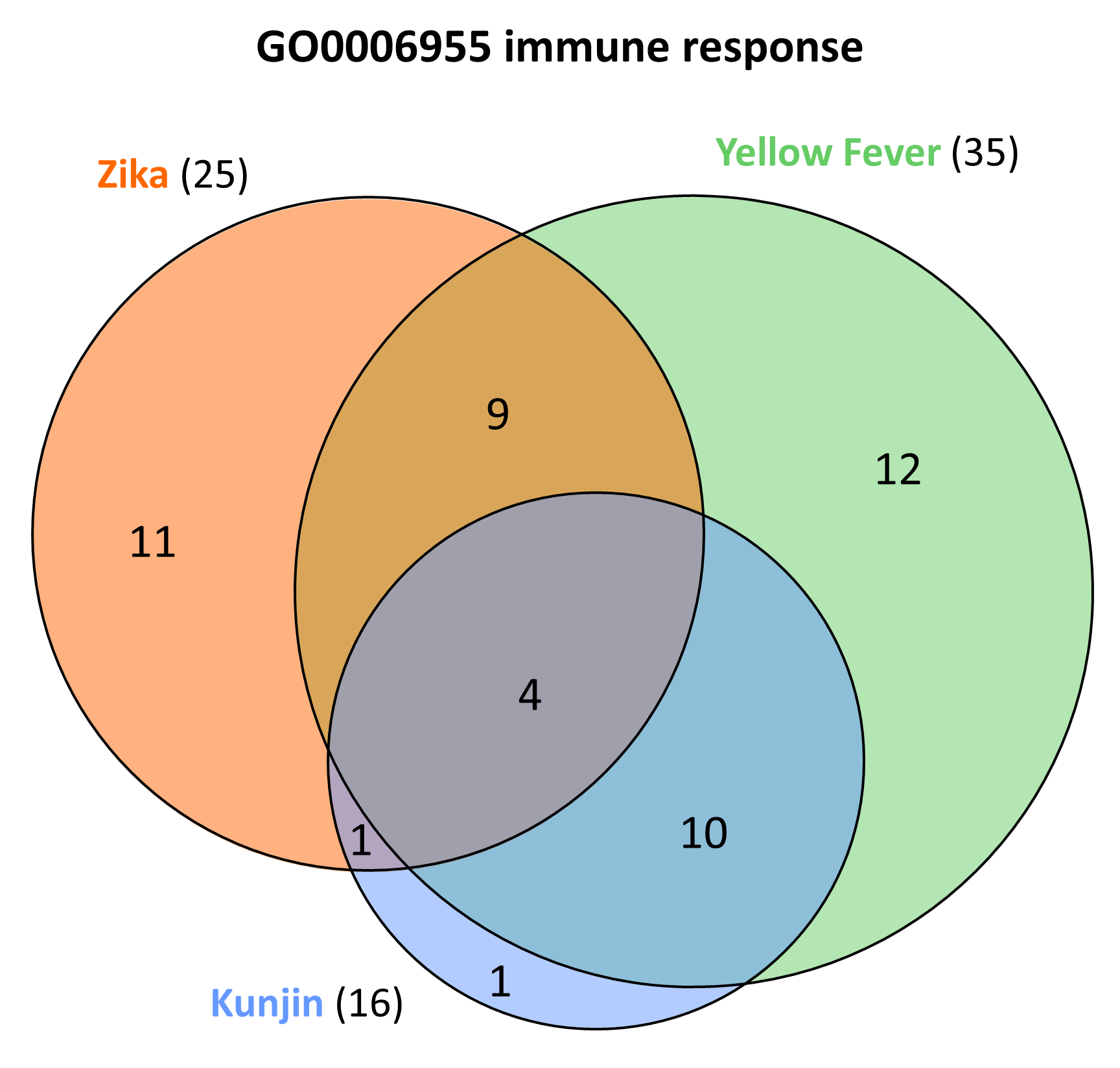

Supplement: Supplementary file 1 [file viruses-15-01419-s001.zip › FigureS5.TIF]

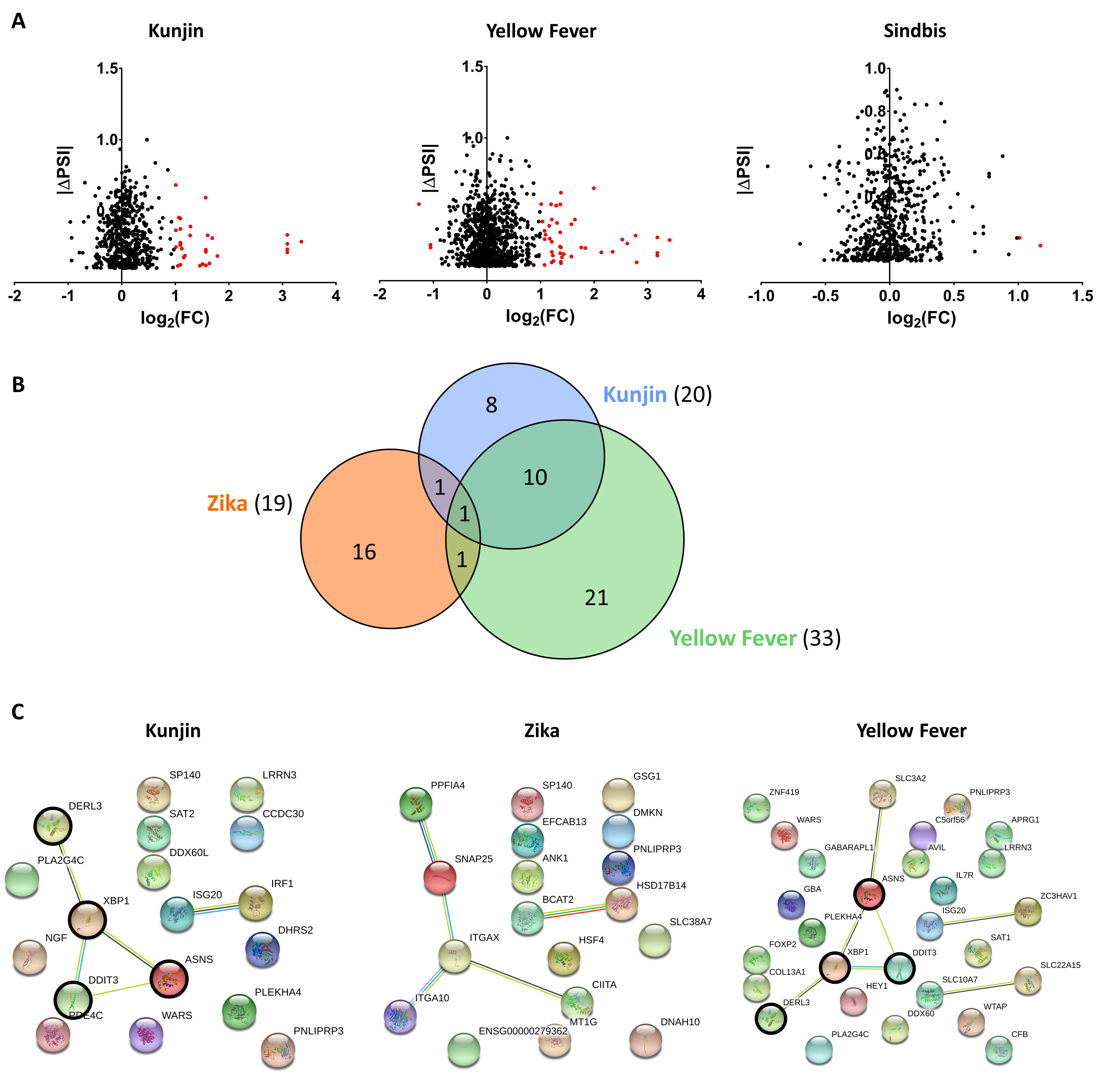

Supplement: Supplementary file 1 [file viruses-15-01419-s001.zip › FigureS6.TIF]

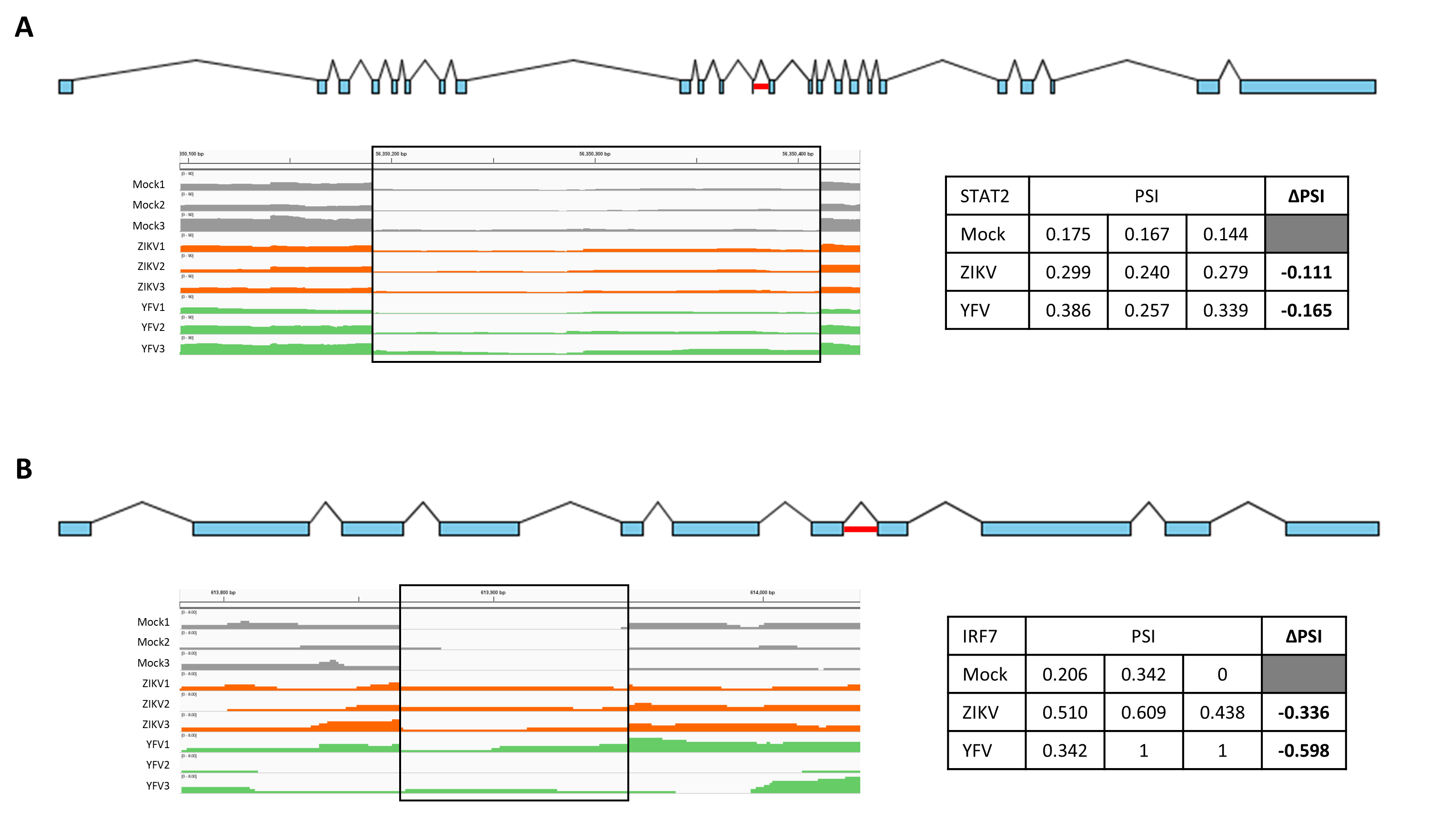

Supplement: Supplementary file 1 [file viruses-15-01419-s001.zip › FigureS7.TIF]

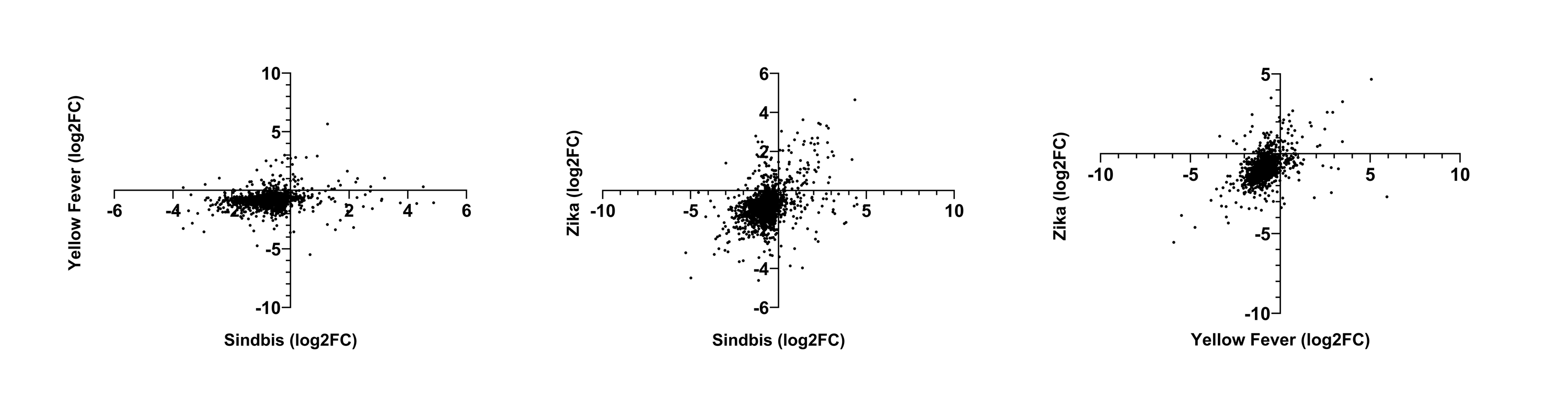

Supplement: Supplementary file 1 [file viruses-15-01419-s001.zip › FigureS8.TIF]

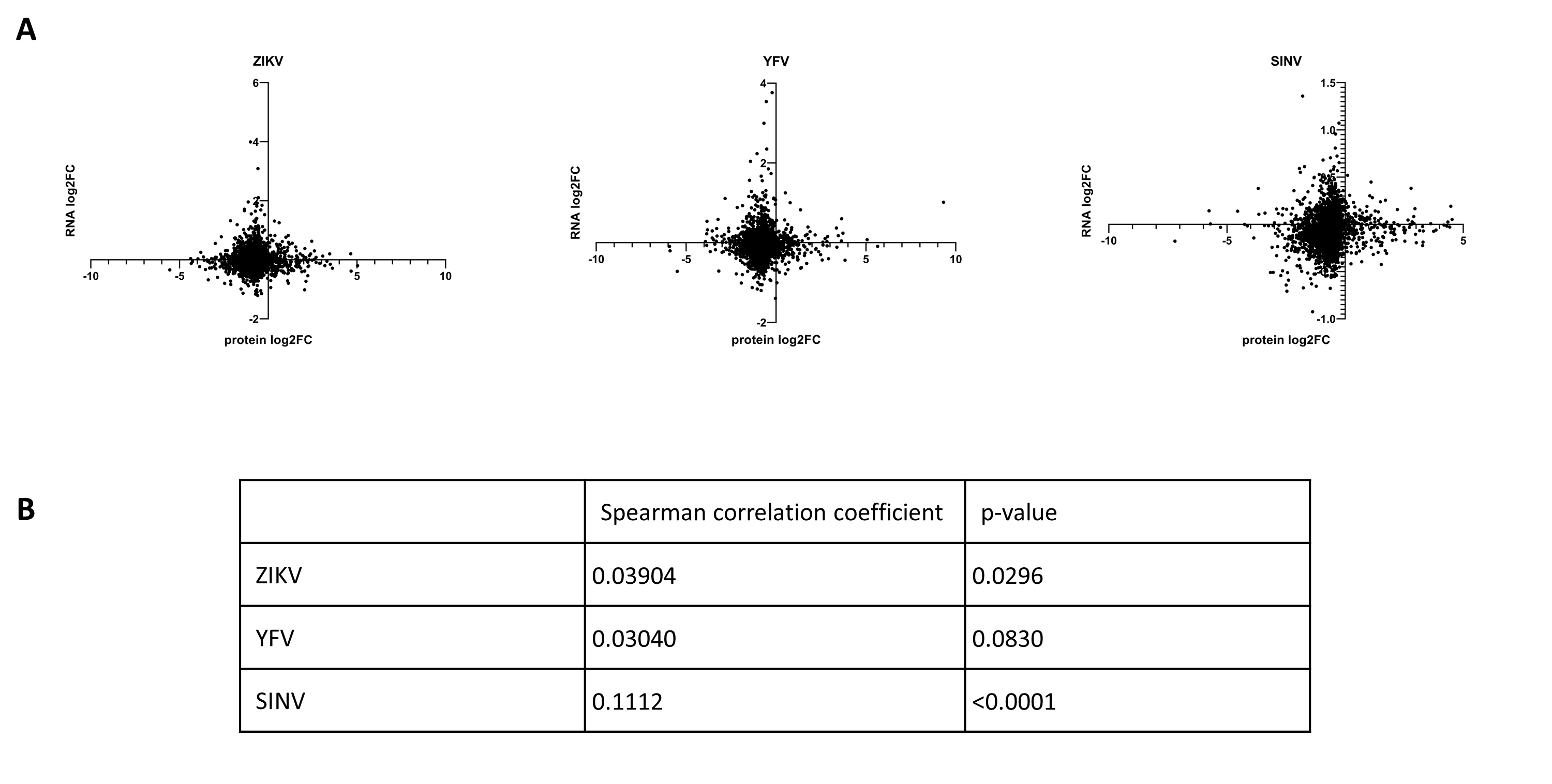

Supplement: Supplementary file 1 [file viruses-15-01419-s001.zip › FigureS9.TIF]
